# “Feeling It”: Links between elements of compassion and sexual well-being

**DOI:** 10.3389/fpsyg.2022.1017384

**Published:** 2023-01-04

**Authors:** Ashley M. Fraser, Chelom E. Leavitt, Jeremy B. Yorgason, Amber A. Price

**Affiliations:** School of Family Life, Brigham Young University, Provo, UT, United States

**Keywords:** compassion, mindfulness, sex, relationships, dyadic

## Abstract

**Introduction:**

Compassion may be a particularly important component of a sexual relationship as it facilitates needed self-awareness, understanding, and connection to frame deeply intimate expressions of sexual emotion and vulnerability. Given the lack of research on how broad concepts of compassionate elements may be linked to sexual well-being, we examine how mindfulness (an ability to maintain awareness in the present moment), compassionate relational attitudes (i.e., accessibility, responsiveness, and engagement), and compassionate relational behaviors (i.e., forgiveness and gratitude), are linked to sexual well-being (sexual harmony, orgasm consistency, and sexual frequency), and sexual mindfulness (a state of being mindful during sex) for oneself and one’s partner.

**Methods:**

We constructed an actor partner structural equation model with newly married couples (*n* = 2,111) and regressed sexual outcomes at time 1 and time 2 on each partner’s compassionate attitudes, behaviors and mindfulness reported at time 1.

**Results:**

Results showed that cross-sectionally, nearly all elements of one’s compassion related to one’s own sexual well-being for both partners. Strongest paths included positive significant relations for women between mindfulness and non-judgment and from compassionate relational attitudes and behaviors to sexual harmony. Men’s compassionate behaviors were positively related to their own sexual awareness. Perhaps more importantly, women’s and men’s compassionate behaviors had significant effects on their partner’s sexual well-being longitudinally.

**Discussion:**

Implications include an emphasis on compassion as a key mechanism that can increase sexual satisfaction and strengthen relationships, particularly in the critical time of early marriage where patterns of interconnectedness are being established.

## Introduction

Compassion is inherently relational. Previous literature has focused on compassionate responding in the individual or compassion development in children and youth (see Seppälä et al., 2017). Research and theory on the formation and expression of compassion within a romantic relationship has focused on self-compassion and has shown a number of positive associations (Lathren et al., 2021). However, researchers have suggested that compassion may be multi-dimensional and include elements such as kindness, common humanity, mindfulness (an ability to maintain awareness in the present moment), and a lack of indifference toward others (Hoisington, 2013; Pommier et al., 2020). These “elements of compassion” are likely essential within early marriage as individuals try to navigate the assumptions and standards created for a romantic relationship (i.e., first five years; Fincham et al., 2006). Compassion within the relationship transcends the individual and thereby facilitates a cohesion between partners that perpetuates relational health. A key piece of relational health in a marriage is sexual well-being, a physiological system that can be activated in response to compassionate partnering, and in turn, strengthen romantic relationships on the whole.

We use the Developmental Model of Marriage Competence (DMMC) as a grounding model for the study (Allsop et al., 2021). Carroll et al. (2006) outline how DMMC is comprised of three key factors that promote formation and maintenance of healthy marital relationships—other-centeredness, personal security, and effective negotiation. Other-centeredness is a particularly salient component and may be especially needed early on in marriage. Compassion is other-centered and will likely add to the development of marriage competence (Carroll et al., 2006) and as Karremans et al. (2017), compassion may also create an other-connectedness in conjunction to other-centeredness. Some other constructs such as forgiveness, gratitude, attachment and mindfulness could also overlap strongly with the elements of personal security (i.e., attachment, mindfulness) and effective negotiation (i.e., forgiveness, gratitude) in a romantic relationship. Thus, in the current study we take a similarly multidimensional view of the factors that contribute to compassionate relational responding and conceptualize such a framework as *relational compassion.*

### A framework of relational compassion

Compassion may be defined as an “awareness of the suffering of another coupled with the wish to relieve it” and may also include an “unselfish concern for the welfare of others” (Marriam-Webster Online Dictionary, n.d.). Synonyms for compassion include condolence, pity, empathy, commiseration, leniency, and tolerance. In discussing the landscape of compassion definitions and approaches in the social sciences specifcally, Goetz and Simon-Thomas (2017) further described distinct processes of compassion including (1) an awareness of need in another person, (2) feeling “moved,” or having physiological response, (3) appraisal of one’s own social role within the context, (4) judgment about the person suffering within the context, and (5) drive to engage in caregiving or helping. As noted, compassion likely moves beyond simple awareness or concern within committed romantic relationships because it is embedded within a context where interaction maintains commitment and connection over time between two people. Thus, a more complex *relational* definition of compassion likely embodies mindfulness, as well as relational attitudes and behaviors that align with Goetz and Simon-Thomas’ processes.

In the current study, we use the phrase *relational compassion* as an umbrella term that encompasses compassionate “elements” that relate specifically to romantic relationships. Considering the complexity of what compassion within a romantic relationship (as opposed to self-compassion or generalized compassion) might look like, we operationalize *relational compassion* as the broad, multidimensional use of personal mindfulness (i.e., ability to be fully aware in the present moment), compassionate attitudes (e.g., relational accessibility) and compassionate behaviors (e.g., forgiveness). Recent measurement work on compassion has included the development of scales that measure individuals’ compassion specifically (e.g., Raes et al., 2011; Pommier et al., 2020). However, in efforts to expand the ways that compassion might be operationalized in a relationship context, we grouped compassion-adjacent constructs together that represented psychological systems (e.g., attitudes, mindfulness) as well as behavioral systems unique to a relational environment (e.g., forgiveness and gratitude) in line with the DMMC. The resulting constructs could facilitate positive sexual responding, which could additionally be considered *compassionate* responding in that sexual relations are inherently dyadic, vulnerable, and take a great deal of thought and care when done well.

We configured these “groupings” in such a way that each represented a concrete piece of relational compassion that could be improved through intentional, guided effort on the part of the individual or couple. Although working on forgiveness, *per se*, does not exactly mean that an individual is increasing their overall compassion, it could contribute to compassionate responding, or even a compassionate relational outlook/framework within their relationship that then leads to other positive relational outcomes (i.e., sexual well-being). Although each construct might predict sexual well-being individually, the three elements combined can represent a relational mindset or framework (i.e., relational compassion) where the whole is theoretically more than the sum of its parts.

Sexual arousal and well-being represent physiological systems in the body that are sensitive, reactive, and bring pleasure and joy to individuals and couples. Sharing in sexual expression with a caring partner can build trust and strengthen the bonds between two people (Leavitt et al., 2021a). Relational compassion is likely inherent to such sexual processes as a precursor, an outcome, and potentially an interactive thread that weaves two people together through sexual harmony. However, these relations have never been theoretically or empirically explored. Thus, building on separate, but equally compelling bodies of work, this study will use a novel approach to examine the links between relational compassion and comprehensive measures of sexual well-being.^1^ Researchers have called for more comprehensive assessments of sexual well-being that do not rely on unidimensional sexual satisfaction measures (e.g., McClelland, 2010; Leavitt et al., 2021c). In response, we include orgasm consistency, sexual frequency, sexual satisfaction, sexual awareness and non-judgment, and sexual harmony as outcomes in the present study. This expansion in understanding can illuminate how specific elements of relational compassion might be bolstered to facilitate healthier, lasting relationships by way of sexual well-being.

### Relationally compassionate elements

#### Mindfulness

Mindfulness may be one component of compassion that is targeted and supportive of emotional development to alleviate suffering (Germer, 2009; Goetz and Simon-Thomas, 2017). Mindfulness is an ability to remain aware and mentally present in a given moment (Kabat-Zinn, 1990). Being aware has shown a host of positive links with relational and sexual well-being (Harvey et al., 2019; Eyring et al., 2021: Leavitt et al., 2021a). This positive association is likely found because mindfulness plays an important role in creating connection. Karremans et al. (2017) provide a theoretical framework explaining that mindfulness within a relationship helps individuals reevaluate interactions and make more positive assessments of those interactions. Being more aware also helps romantic partners slow down their thought process so their decisions can be more intentional and less reactive. Mindful awareness also helps romantic partners to create a stronger self-other connection, or in other words, awareness facilitates an understanding of how others’ responses are influenced by external circumstances. Mindful individuals can take their partner’s perspective. In a one-year longitudinal study using adolescents, young people who learned mindfulness techniques later reported increases in overall mindfulness, self- and other-compassion (Stutts et al., 2018). This response aligns with the DMMC (Carroll et al., 2006) as well as Goetz and Simon-Thomas (2017) processes of compassion. Mindful individuals can be more personally secure and intentional about the connections created within the relationship to account for partner preferences and strengthen the marriage.

#### Relationally compassionate attitudes: Accessibility, responsiveness, and engagement

Attitudes of attachment such as accessibility, responsiveness, and engagement capture elements of compassion between partners. Mikulincer and Shaver (2005) found that these attitudes are foundational to compassionate caregiving whereas relational insecurities interfere with compassionate responding. The presence of physical, emotional, and psychological accessibility (being available) or responsiveness (awareness and sensitivity) from a partner may help the individual endure stress and uncertainty or gain confidence needed for growth and learning (Johnson, 2003). It’s important to note that accessibility and responsiveness alone are not enough to create a secure relationship. Creating critical bonding moments that are described as engagement are also essential (Sandberg et al., 2012). If a partner can request closeness or connection and rely on its occurrence, a new bonding experience occurs (Johnson, 2003). This third marker of engagement rounds out the aspects of positive attitudes that align with relational compassion (Johnson, 2003; Sandberg et al., 2012). These attitudes likely create a context that is other-centered and other-connected (Carroll et al., 2006; Goetz and Simon-Thomas, 2017; Karremans et al., 2017), through engaging in responsive conversation that sees the individual’s needs and desires.

#### Relationally compassionate behaviors: Forgiveness and gratitude

Forgiveness is a dispositional tendency that may affect an individual’s intrapersonal well-being and relationships (Berry and Worthington, 2001). Forgiveness is defined as strong, positive, other-oriented emotions that supersede the negative emotions of unforgiveness (Worthington and Wade, 1999), and while transgressions are bound to be a part of any relationship, compassion through forgiveness may play an important role. Neff (2003) has suggested that the mechanism through which self-compassion works is not that painful feelings are avoided but instead that an awareness including kindness, understanding, and a sense of shared humanity is adopted. In fact, compassion and forgiveness seem to work hand-in-hand as individuals work to overcome trauma (Ghasem Zadeh et al., 2019; Erskine, 2020). Forgiveness may also help couples work through challenges in their sexual relationship.

Gratitude may also be a behavior that is expressed within a compassionate framework. Kabat-Zinn (1990) described the heartfelt side of mindfulness as “appreciative” and “nurturing,” or “heartfelt.” Gratitude and compassion have been paired in describing this heartfelt side of mindfulness (Voci et al., 2019). Both gratitude and compassion were found to be mechanisms through which mindfulness linked to psychological outcomes such as positive relations with others and purpose in life (Voci et al., 2019). Gratitude has shown both direct and indirect (through increased empathy) connections with compassionate love (Kim et al., 2018) as individuals with higher levels of gratitude showed higher levels of compassion by way of being more empathetic. In line with the DMMC, these behaviors may be key to effective negotiation in the relationship as both gratitude and forgiveness represent exchanges where couples agentically offer thanks to their partner and potentially offer grace for mistakes as well (Carroll et al., 2006; Karremans et al., 2017).

### Markers of sexual well-being

Sex is both physical and emotional and therefore needs to be examined in a way that captures the multifaceted nature of the experience, particularly for women (Kleinplatz et al., 2009; McClelland, 2010). To accomplish this, we examine sexual harmony, orgasm, sexual frequency, and the two components of sexual mindfulness: awareness and non-judgment as indicators of overall sexual well-being.

#### Sexual harmony

Sexual harmony builds on the theory of general passion (Vallerand, 2010) and is evidenced by a sexual interest that is not fleeting but instead a core part of a couple’s identity and life satisfaction (Busby et al., 2019). Researchers have suggested that standing in contrast to an obsessive or inhibited sexual attitude is harmonious sexual passion (Philippe et al., 2017). Sexual harmony is a balanced, self-directed, and controlled commitment for sex, which leads to positive individual and relational outcomes (Philippe et al., 2017; Busby et al., 2019). Sexual harmony is a balance of sexual needs and attitudes that are in harmony with the holistic relationship. This balance is demonstrated in a longitudinal study that found an intricate bi-directional association of relational and sexual well-being, indicating that both influence each other (McNulty et al., 2016).

#### Orgasm consistency

Although orgasm is less consistent for women than men, orgasm is important for both to achieve sexual well-being (Leavitt et al., 2021b). Because orgasm is described as the pinnacle of sexual pleasure, it is often used as an indicator of sexual well-being, as well as sexual competence and sexual satisfaction (Haning et al., 2007; Young et al., 1998; Potts, 2000; Komisaruk et al., 2009). Orgasm has been linked with self-compassion such that individuals with greater self-compassion are likely to report greater orgasm consistency and husbands’ self-compassion may safeguard against negative effects of distress about sexual problems for both their own sexual satisfaction and their partners’ (Ferreira et al., 2020).

#### Sexual frequency

Although research often connects sexual frequency with sexual satisfaction (Sánchez-Fuentes et al., 2014), some research indicates these measures may not reveal a complete picture of sexual satisfaction for women (Brotto, 2010). Sexual frequency is certainly important for both men’s and women’s sexual well-being (Frederick et al., 2017), but maybe not as linearly associated as some have suggested (Muise et al., 2016). Muise and colleagues found that the association between frequency and well-being is curvilinear not linear, and sex was not associated with well-being for frequencies more than once a week. So, while sexual frequency is important, it may not always have a linear relationship with sexual well-being.

#### Sexual mindfulness

As noted, sexual relationships are complex and often filled with additional anxieties due to sharing naked bodies, performance issues, or self-criticism. Sexual mindfulness is a skill built off trait mindfulness, but applied within a sexual context (Leavitt et al., 2019). Not all mindful individuals are able to maintain their mindfulness within the context of heightened anxiety and pleasure of sex. Trait mindfulness certainly contributes to sexual mindfulness but it is not sufficient to achieve sexual mindfulness (Leavitt et al., 2019). Additionally, recent research has shown the significant effects of sexual mindfulness in improving sexual communication, connectedness, sexual functioning, and sexual satisfaction (Leavitt et al., 2021a,e). Consequently, we use the state of achieving sexual mindfulness as a component of sexual well-being that likely derives from the multi-dimensional elements of relational compassion.

##### Sexual mindful awareness

Leavitt et al. (2019) found that being aware during a sexual experience was associated with sexual satisfaction, as well as relational satisfaction and self-esteem, above and beyond mindfulness alone. Other research has shown that sexual mindful awareness was associated with the individual’s and their partner’s orgasm consistency, sexual harmony, and relational flourishing (Leavitt et al., 2021c). Being aware during sex likely creates a greater sense of the details surrounding the sexual experience and as Karremans et al. (2017) explain, mindfulness encourages a higher level of executive function, increases emotion regulation, as well as self-other connectedness. This type of presence fosters quality relationships, particularly within a complex sexual relationship. Self-interested and retaliatory impulses are less emphasized as a mindful individual engages in constructive efforts of broader relationship concerns and is attuned to the interests of their partner (Karremans et al., 2017).

##### Sexual mindful non-judgment

Non-judgment is the second component of sexual mindfulness (Leavitt et al., 2019). To be non-judgmental, an individual refrains from criticism or negative evaluations during sex. Instead of judgment, the individual can practice curiosity or observation. When conflict arises during sex, the mindful individual can note differences as just that, differences. There is no need to make evaluations that result in pitting partners against one another. Instead of the evaluation, “My partner doesn’t care about my pleasure,” the individual may be curious about why their partner is disengaged or distracted. These non-evaluative observations allow couples to further investigate differences and find common ground or aligned sexual interests (Rogers, 2016).

### Relational compassion and sex

Each relationally compassionate element discussed above can be connected to various aspects of sexual well-being. Though no research we know of has linked *relational compassion* and sexual well-being generally, some “sister” research has evaluated the role of *self*-compassion within circumstances of sexual distress and found that self-compassion is negatively associated with sexual distress, but not necessarily with sexual satisfaction (Santerre-Baillargeon et al., 2018; Michael et al., 2021). Other studies found positive links between mindfulness and sexual well-being (Leavitt et al., 2021c) and compassion and to higher relationship quality generally (McDonald et al., 2020). The present study aims to expand this literature to encompass more comprehensive measures of both compassion and sexual well-being to expand understanding of a dynamic process and promote healthy romantic relationships.

First, mindfulness may contribute to sexual well-being through multiple avenues. Theoretical as well as empirical work indicates that mindfulness contributes to a general sense of compassion toward others in the form of perspective taking (Lim et al., 2015; Karremans et al., 2017, 2020). Being personally aware and fully “present” in a relational context can surely contribute to both sexual frequency and orgasm frequency as each partner stays intimately attuned to the others’ physical needs, responding to sexual requests and reacting compassionately to their partner’s body through high-quality sexual communication and physical responsiveness. The ability to take another’s perspective can enhance awareness of each partner’s sexual needs and desires, which can contribute to mutually satisfactory sex that prizes vulnerability, repels self- and partner-judgment, and helps the couple align both physically and emotionally (e.g., sexual harmony). The “shared humanness” aspect encompassed in personal mindfulness may also help a couple relate to one another emotionally during sex as they share a physical and psychological bond.

Secondly, couples who struggle to compassionately relate and respond to their partner (i.e., negative relationally compassionate attitudes) may also show negative sexual patterns. Notably, unhealthy couple dynamics that are anxious or avoidant are associated with lower sexual satisfaction (Busby et al., 2020). However, less is known about how relational attitudes are related to other important dimensions of sexual well-being, which we explore in the present study. Unknowns aside, it makes theoretical sense that couples who are psychologically accessible are more likely to respond positively to requests for sex, or for certain sexual behaviors from one’s partner. This accessibility, coupled with responsiveness and engagement, likely provides a safe space where each partner can relate to the other in intimate, physical ways that contribute to sexual satisfaction, harmony, and a consistently pleasurable and unifying sexual experiences. Using our underlying theoretical understanding of other-centeredness, other-connectedness, and compassion (Carroll et al., 2006; Goetz and Simon-Thomas, 2017; Karremans et al., 2017), the present study explores these hypotheses.

Finally, forgiveness as an element of relational compassion may be particularly needed in sexual relationships as sex represents an intimate expression of complex emotion and vulnerable areas of our identity (Kleinplatz et al., 2009). Indeed, it could be that when couples are not “on the same page” about sexual frequency, sexual behaviors, or the sexual dissatisfaction of one or both partners, it is the compassionate ability to forgive that sustains a healthy sexual relationship over time. Couples who can forgive one another likely demonstrate more sense of sexual communal strength, which is an awareness and desire to meet the sexual needs of each other despite challenges (Muise et al., 2016), and is associated with sexual satisfaction. In support of this supposition, men and women who were more forgiving showed a positive connection for not only their own sexual well-being but also for their partner’s sexual well-being (Eyring et al., 2021).

Additionally, gratitude has shown a positive association with not only an individual’s sexual well-being, but their partner’s sexual well-being (Eyring et al., 2021). This is unsurprising as gratitude is complicit with nurturance, unconditional acceptance, deep connection and appreciation. Each of these qualities is sure to facilitate a positive sexual relationship in which partners can express their needs, respond to the other with sympathy and/or gentleness, and work through difficult sexual issues with understanding (e.g., past traumas, infertility concerns, performance concerns). Both forgiveness and gratitude (i.e., relationally compassionate behaviors) could potentially lead to increases in sexual frequency, sexual satisfaction and orgasm consistency, as well as heightened sexual awareness, non-judgment and harmony as both create a context of other-centeredness that likely benefits the early patterns of marriage formation (Carroll et al., 2006; Karremans et al., 2017).

### The current study

Given the lack of research on how broad concepts of relational compassion (as opposed to self-compassion or generalized compassion) may be linked to sexual well-being, we examine attitudes and behaviors that are elements of relational compassion to evaluate their links to sexual harmony, sexual frequency, orgasm consistency, and sexual mindful awareness and non-judgment cross-sectionally (time 1; T1) and over time (time 2; T2). We do so in an actor-partner model such that we can explore ways that relationally compassionate elements influence an individual’s own, as well as their partner’s, sexual well-being concurrently and 2 years later. Based on previous literature, we hypothesized:

H1: Husbands’ and wives’ relational compassion (mindfulness, compassionate attitudes, and compassionate behaviors), would be positively associated with one’s own sexual harmony, sexual frequency, orgasm consistency, and sexual mindful awareness and non-judgment both within and across time.

H2: Husbands’ and wives’ relational compassion would be positively associated with their spouses’ sexual harmony, sexual frequency, orgasm consistency, and sexual mindful awareness and non-judgment both within and across time.

## Materials and methods

### Sample

Participants for this study were drawn from a larger nationally representative longitudinal study of positive relationship interactions and virtues during the beginning (first five) years of marriage (*N* = 2,111 couples). Data was initially collected in October of 2015 and the entire sample for the present study was composed of heterosexual couples who had married in 2013 (4%), 2014 (90%), and 2015 (6%) (see James et al., 2022). The majority of couples in the present study had been married approximately two years in Time 1 of our study and four years at Time 2 of our study. The data was dyadic such that participants were married to one another. For the current study, the average age for wives at T1 was 28.0 years old (*SD* = 5.1) and the average age for husbands at T1 was 29.85 years old (*SD* = 5.64). Race/ethnicity of participants included: White (66% wives, 65% husbands), Black/African American (9% wives, 11% husbands), Hispanic/Latino (13% wives, 13% husbands), Asian (5% wives, 3% husbands), Multiracial (6% wives, 6% husbands), and Other (1% wives, 2% husbands). Educational attainment included: less than high school (2% wives, 4% husbands), high school (14% wives, 22% husbands), some college (28% wives, 29% husbands), Associate’s degree (12% wives, 10% husbands), Bachelor’s degree (29% wives, 25% husbands), Master’s degree (11% wives, 7% husbands), advanced degree such as Ph.D. or J.D. (4% wives, 4% husbands).

### Measures

All scale scores below were standardized into z-scores (outside of control variables) before being entered into the structural equation model. All measures were self-reported.

#### Mindfulness

Mindfulness at T1 was measured using the Mindful Attention Awareness Scale (15 items; Brown and Ryan, 2003). Items are on a Likert-type scale ranging from 1 (almost always), to 6 (almost never), with higher scores indicating higher state mindfulness (e.g., “I find myself listening to someone with one ear, doing something else at the same time”). The scale had adequate reliability in this sample for both wives and husbands (Chronbach’s αs = 0.85,0.86).

#### Compassionate attitudes

Compassionate attitudes were measured using the Brief Accessibility, Responsiveness, and Engagement (BARE) scale (Sandberg et al., 2012). Respondents responded to statements regarding their personal accessibility, responsiveness, and engagement with their partner on a 12-item scale from 1 (never true) to 5 (always true). Example items included, “I am confident I reach out to my partner,” and “My partner listens when I share my deepest feelings.” The scale had adequate reliability in this sample for both wives and husbands (Chronbach’s αs = 0.78,0.80), with higher scores indicating higher levels of compassionate attitudes.

#### Compassionate behaviors

Compassionate behaviors included a mean-composite score of each partner’s forgiveness and gratitude, where higher scores indicated higher levels of compassionate behavior. Six items came from the Marital Forgiveness Scale and three came from the Gratitude Scale (Fincham et al., 2006; Lambert et al., 2009). Sample items included, “I soon forgive my partner,” and “When my partner does something nice for me I acknowledge it.” The combined scale had adequate reliability in this sample for wives and husbands (Chronbach’s αs = 0.83,0.84).

#### Sexual frequency

Each partner reported how often they currently had sex with their partner in a single item at T1 and T2. Responses included: 1 = Never, 2 = Less than once a month, 3 = One to three times a month, 4 = About once a week, 5 = Two to four times a week, 6 = Five to seven times a week, 7 = More than once a day.

#### Sexual satisfaction

Each partner reported how satisfied they were with how often they were having sex in a single item at both timepoints. Responses ranged from 1 = very dissatisfied to 5 = very satisfied.

#### Orgasm consistency

Participants reported how often they experienced orgasm when they were sexual with their partner (1 = 0–20% of the time, 2 = 21–40% of the time, 3 = 41–60% of the time, 4 = 61–80% of the time, 5 = 81–100% of the time) at T1 and T2.

#### Sexual harmony

Each partner responded to three items from the Harmonious subscale of the Sexual Passion Scale (adapted from Vallerand, 2010; Lalande et al., 2017). Responses ranged from 1 = never to 5 = very often with higher scores indicating higher harmony. A sample item included, “The sexual activities that I am excited about in my relationship with my partner are in harmony with other things that are a part of me.” The scale had adequate reliability in this sample for both wives and husbands at T1 (Chronbach’s αs = 0.91,0.90) and T2 (Chronbach’s αs = 0.91,0.90).

#### Sexual awareness

Sexual awareness was assessed using a subscale of the Sexual Mindfulness Measure developed by Leavitt et al. (2019). Participants responded to four items, such as “I pay attention to how sex affects my thoughts and behavior,” on a one to five scale ranging from “Never or Rarely True” to “Very Often or Always True.” The scale had adequate reliability for wives and husbands at T1 (Chronbach’s αs = 0.85,0.82) and T2 (Chronbach’s αs = 0.85,0.82).

#### Sexual non-judgment

Respondents reported their non-judgment by responding to three items, another subscale of the Sexual Mindfulness Measure (Leavitt et al., 2019), that asked how judgmental they were during sex on a 1 (never or rarely true) to 5 (very often or always true) scale, which were then reverse coded (e.g., “During sex, I sometimes get distracted by evaluating myself or my partner”). The scale had adequate reliability for wives and husbands at T1 (Chronbach’s αs = 0.85,0.83) and T2 (Chronbach’s αs = 0.84,0.82).

#### Relevant controls

Control variables included participant age (computed from birthdate), education level, and race (response items can be seen in the Participants section). Age can be relevant as sexual frequency, satisfaction and orgasm consistently certainly change across age, even from the span of early to late twenties, particularly for women (Hayes and Dennerstein, 2005). This may be due to childbirth, hormonal fluctuations, and career advancement/change, and other factors. Race is additionally relevant as we know that marital dynamics, and in conjunction, sexual dynamics, differ across context and culture (see debates stemming from Rushton and Bogaert, 1987 to present, e.g., Bharj, 2020).

### Procedures

Participants were recruited across a large, nationally representative sample of over 2,000 young married couples in the United States beginning in fall 2015. Participants were recruited using a two-stage cluster stratification sample design. The first stage involved a sample of US counties, and the second involved a sample of recent marriages within them. Selection was based on county population size, marriage, divorce, poverty rates, and the racial-ethnic distribution of the county. Initially, potential participants were contacted by mailed letters that contained a $2.00 bill with an invitation to participate and instructions on how to enroll in the study. Subsequently, follow-up postal mailings, e-mail invitations, and phone calls were made. Those that opted-in to the study were directed to an online Qualtrics survey. Participating couples were given a $50.00 Visa gift card upon completion of the survey. The study was approved by all appropriate IRB bodies.

### Analysis plan

**FIGURE 1:**
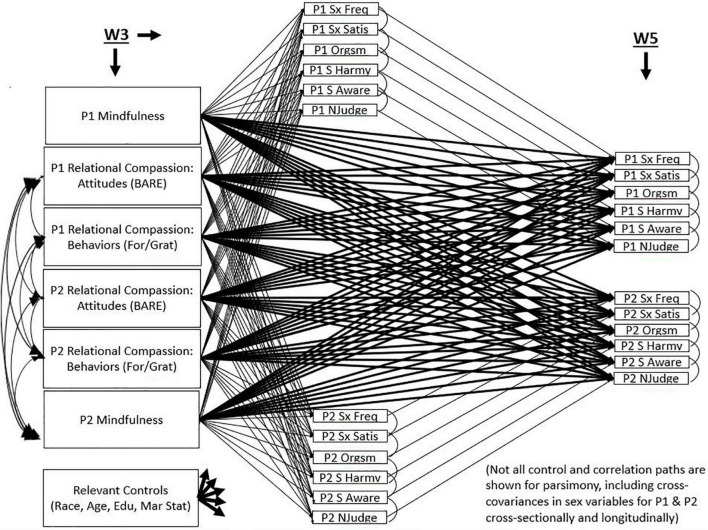
Longitudinal actor partner interdependent model showing cross-sectional and longitudinal relations between elements of relational compassion and sexual wellbeing across two years.

All variables were assessed for distributional normality in SPSS 28. Pearson’s correlations between all variables were estimated to check for collinearity among predictors in the model. Additionally, one-way ANOVAs and correlations were estimated to determine which control variables should be included in the main analysis. After this was determined, a longitudinal cross-lagged actor-partner model (APIM) was constructed in MPLUS v.8 where T1 and T2 outcome variables (sexual frequency, sexual satisfaction, orgasm consistency, harmony, awareness, and non-judgment) for each partner were regressed on T1 predictors (mindfulness, compassionate attitudes, compassionate behaviors) for each partner, as well as relevant controls (see Figure 1). Additionally, T2 outcomes were regressed on T1 outcomes to control for stability in sexual well-being constructs across time. The typical actor-partner independence model (APIM) explores associations between predictors from both partners predicting own and partner outcomes (Cook and Kenny, 2005). We used the APIM framework to explore concurrent and 2-year longitudinal associations between predictors and outcomes. Although some researchers would correlate predictors and outcomes concurrently and explore predictions across time, because the associations we were examining had important concurrent associations as well as longitudinal ones, we predicted outcomes at the same timepoint as well as 2 years later, as has been done in previous research (Cook and Kenny, 2005; Kenny et al., 2006; Pollock Star et al., 2022). Stability paths were included such that T2 outcomes were controlled for at T1. This model allowed us to (1) assess the relative strength of paths from each predictor to each outcome when considered in concert with the other predictors, (2) compare actor effects to partner effects on sexual well-being, and (3) view actor and partner relations between compassionate constructs and sexual well-being both cross-sectionally and longitudinally 2 years later.

## Results

### Preliminary analyses

Correlations and descriptive statistics including distributional properties for all variables are in Table 1. All correlations were in the expected direction.

**TABLE 1:** Correlations and descriptive statistics for all study variables (*n* = 2,177).

	1.	2.	3.	4.	5.	6.	7.	8.	9.	10.	11.	12.	13.	14.	15.
1. P1 mindfulness	1														
2. P1 compassionate attitudes	0.37***	1													
3. P1 compassionate behaviors	0.23***	0.51***	1												
4. P2 mindfulness	0.24***	0.18***	0.12***	1											
5. P2 compassionate attitudes	0.18***	0.45***	0.31***	0.35***	1										
6. P2 compassionate behaviors	0.13***	0.32***	0.29***	0.22***	0.58***	1									
7. P1 T1 sexual frequency	0.10***	0.17***	0.15***	0.10***	0.14***	0.10***	1								
8. P1 T1 sexual satisfaction	0.20***	0.24***	0.21***	0.16***	0.20***	0.16***	0.58***	1							
9. P1 T1 orgasm consistency	0.15***	0.25***	0.20***	0.08**	0.16***	0.13***	0.24***	0.26***	1						
10. P1 T1 sexual harmony	0.18***	0.36***	0.31***	0.11***	0.27***	0.20***	0.45***	0.47***	0.35***	1					
11. P1 T1 sexual awareness	0.08**	0.19***	0.20***	0.04	0.13***	0.11***	0.25***	0.15***	0.28***	0.46***	1				
12. P1 T1 sexual non-judgment	0.30***	0.30***	0.19***	0.15***	0.19***	0.11***	0.05+	0.13***	0.19***	0.23***	−0.01	1			
13. P2 T1 sexual frequency	0.08**	0.13***	0.13***	0.11***	0.19***	0.15***	0.70***	0.44***	0.19***	0.39***	0.22 ***	0.08**	1		
14. P2 T1 sexual satisfaction	0.13***	0.18***	0.18***	0.21***	0.26***	0.20***	0.44***	0.43***	0.20***	0.37***	0.18 ***	0.13***	0.58***	1	
15. P2 T1 orgasm consistency	0.05+	0.15***	0.10***	0.08**	0.18***	0.12***	0.11***	0.06+	0.14***	0.11***	0.13 ***	0.06+	0.15***	0.11***	1
16. P2 T1 sexual harmony	0.09**	0.22***	0.21***	0.18***	0.35***	0.28***	0.34***	0.28***	0.20***	0.45***	0.25 ***	0.14***	0.45***	0.47***	0.06*
17. P2 T1 sexual awareness	0.01	0.12***	0.13***	0.06+	0.20***	0.23***	0.14***	0.10***	0.14***	0.19***	0.21 ***	−0.02	0.17***	0.07*	0.11***
18. P2 T1 sexual non-judgment	0.11***	0.19***	0.13***	0.27***	0.35***	0.23***	0.07*	0.08**	0.07**	0.10**	−0.03	0.19***	0.08**	0.08**	0.20***
19. P1 T2 sexual frequency	0.11***	0.12**	0.11***	0.09**	0.14***	0.11***	0.52***	0.33***	0.18***	0.29***	0.13 ***	0.06*	0.46***	0.27***	0.06*
20. P1 T2 sexual satisfaction	0.12***	0.11**	0.11***	0.11***	0.10**	0.11***	0.29***	0.44***	0.13***	0.25***	0.09 **	0.08*	0.24***	0.22***	0.05
21. P1 T2 orgasm consistency	0.11***	0.16***	0.11***	0.04	0.11***	0.10***	0.18***	0.19***	0.69***	0.28***	0.23 ***	0.10***	0.15***	0.16***	0.09**
22. P1 T2 sexual harmony	0.15***	31***	0.25***	0.09**	0.25***	0.20***	0.31***	0.33***	0.31***	0.46***	0.25 ***	0.16***	0.26***	0.26***	0.08*
23. P1 T2 sexual awareness	0.10***	0.15***	0.18***	0.07**	0.08*	0.06*	0.20***	0.13***	0.23***	0.33***	0.48 ***	0.05	0.17***	0.16***	0.03
24. P1 T2 sexual non-judgment	0.24***	0.26***	0.18***	0.10**	0.17***	0.10***	0.01	0.09**	0.18***	0.19***	0.07*	0.48***	0.04	0.08**	0.05
25. P2 T2 sexual frequency	0.09**	0.12***	0.12***	0.13***	0.16***	0.12***	0.46***	0.29***	0.16***	0.27***	0.14 ***	0.08**	0.50***	0.32***	0.06+
26. P2 T2 sexual satisfaction	0.07*	0.14***	0.17***	0.18***	0.18***	0.14***	0.27***	0.25***	0.11***	0.24***	0.15 ***	0.05+	0.32***	0.47***	0.02
27. P2 T2 orgasm consistency	0.06*	0.16***	0.10***	0.08*	0.17***	0.11***	0.17***	0.08**	0.20***	0.12***	0.13 ***	0.09**	0.16***	0.10***	0.48***
28. P2 T2 sexual harmony	0.11***	0.21***	0.17***	0.21***	0.32***	0.25***	0.24***	0.20***	0.15***	0.28***	0.18 ***	0.07*	0.32***	0.34***	0.08**
29. P2 T2 sexual awareness	0.04	0.10***	0.07**	0.11***	0.17***	0.19***	0.14***	0.10**	0.08**	0.12***	0.15 ***	0.00	0.13***	0.06*	0.03
30. P2 T2 sexual non-judgment	0.11***	0.17***	0.11***	0.13***	0.26***	0.18***	0.06+	0.07*	0.05	0.14***	0.04	0.13***	0.05+	0.04	0.20***
Mean (SD)	4.22 (0.96)	4.22 (0.66)	4.82 (0.87)	4.33 (0.98)	4.06 (0.68)	4.89 (0.86)	3.70 (1.21)	3.25 (1.22)	3.61 (1.54)	3.24 (1.02)	3.28 (0.88)	3.73 (0.99)	3.69 (1.23)	3.15 (1.27)	4.71 (0.81)
Min/max	1–6	1–5	1.25–6.67	1–6	1.5–5	1.58–6.30	1–7	1–5	1–5	1–5	1–5	1–5	1–7	1–5	1–5
Skew/kurtosis	−0.22/−0.28	−0.76/ 0.30	−0.29/ −0.34	−0.27/ −0.32	−0.53/ −0.26	−0.33/ −0.54	−0.17/ −0.55	−0.23/ −0.89	−0.62/ −1.16	−0.22/ −0.43	−0.21/ 0.10	−0.58/ −0.23	−0.06/ −0.62	−0.13/ −1.06	−3.20 /10.04

	16.	17.	18.	19.	20.	21.	22.	23.	24.	25.	26.	27.	28.	29.	30.

1. P1 mindfulness															
2. P1 compassionate attitudes															
3. P1 compassionate behaviors															
4. P2 mindfulness															
5. P2 compassionate attitudes															
6. P2 compassionate behaviors															
7. P1 T1 sexual frequency															
8. P1 T1 sexual satisfaction															
9. P1 T1 orgasm consistency															
10. P1 T1 sexual harmony															
11. P1 T1 sexual awareness															
12. P1 T1 sexual non-judgment															
13. P2 T1 sexual frequency															
14. P2 T1 sexual satisfaction															
15. P2 T1 orgasm consistency															
16. P2 T1 sexual harmony	1														
17. P2 T1 sexual awareness	0.31***	1													
18. P2 T1 sexual non-judgment	0.17***	−0.07*	1												
19. P1 T2 sexual frequency	0.24***	0.10***	0.04	1											
20. P1 T2 sexual satisfaction	0.18***	0.10**	0.00	0.54***	1										
21. P1 T2 orgasm consistency	0.16***	0.12***	0.05	0.26***	0.23***	1									
22. P1 T2 sexual harmony	0.30***	0.16***	0.04	0.49***	0.45***	0.43***	1								
23. P1 T2 sexual awareness	0.21***	0.14***	−0.04	0.22***	0.16***	0.27***	0.41***	1							
24. P1 T2 sexual non-judgment	0.10**	−0.02	0.11***	0.12***	0.14***	0.21***	0.29***	0.05	1						
25. P2 T2 sexual frequency	0.31***	0.12***	0.04	0.76***	0.43***	0.20***	0.42***	0.19***	0.11***	1					
26. P2 T2 sexual satisfaction	0.29***	0.02	0.02	0.45***	0.37***	0.17***	0.39***	0.19***	0.10**	0.58***	1				
27. P2 T2 orgasm consistency	0.13***	0.09**	0.21***	0.17***	0.05	0.20***	0.22***	0.08*	0.11**	0.19***	0.12***	1			
28. P2 T2 sexual harmony	0.45***	0.17***	0.17***	0.37***	0.28***	0.20***	0.47***	0.21***	0.17 ***	0.48***	0.49***	0.25***	1		
29. P2 T2 sexual awareness	0.20***	0.42***	0.02	0.19***	0.16***	0.12***	0.21***	0.18***	−0.01	0.19***	0.12***	0.11**	0.30***	1	
30. P2 T2 sexual non-judgment	0.16***	0.07*	0.42***	0.06	0.08**	0.07*	0.15***	−0.05	0.21***	0.10**	0.06*	0.27***	0.26 ***	0.01	1
Mean (SD)	3.32 (0.95)	3.37 (0.82)	4.02 (0.88)	3.53 (1.26)	3.25 (1.17)	4.78 (0.67)	3.50 (1.02)	3.29 (0.87)	3.67 (0.97)	3.52 (1.27)	4.71 (0.78)	3.93 (1.27)	3.51 (0.94)	3.39 (0.79)	4.04 (0.84)
Min/max	1–5	1–5	1–5	1–7	1–5	1–5	1–5	1–5	1–7	1–5	1–5	1–5	1–5	1–5	1–5
Skew/kurtosis	−0.19/ −0.26	−0.20/ 0.07	−0.87/ 0.42	−0.06/ 0.53	−0.26/ −0.81	−3.61/ 13.56	−0.40/ −0.30	−0.13/ −0.06	−0.47/ −0.34	0.01/ −0.63	−3.18/10.14	−1.02/ −0.09	−0.35/ −0.09	−0.10/ −0.12	−0.88/ 0.47

*p < 0.05, **p < 0.01, ***p < 0.001.

### Structural equation model

All parameter estimates including *R*^2^ statistics can be seen in Table 2 and a visual representation of the model can be seen in Figure 1. Results will be organized below by timepoint and partner with significant findings highlighted.

**TABLE 2:** Relations between partner 1 (P1) and partner 2 (P2) relationally compassionate constructs at T1 and sexual well-being for self and partner cross-sectionally (T1) and longitudinally (T2) presented as β (standard deviation).

Outcomes predictors	P1 sexual frequency	P1 sexual satisfaction	P1 orgasm consistency	P1 sexual harmony	P1 sexual awareness	P1 sexual non-judgment	P2 sexual frequency	P2 sexual satisfaction	P2 orgasm consistency	P2 sexual harmony	P2 sexual awareness	P2 sexual non-judgment
**Cross-sectional relations (T1 outcomes)**												
P1 mindfulness	0.03 (0.03)	0.10(0.03)***	0.06(0.03)*	0.05(0.03)*	0.01 (0.03)	0.21(0.03)***	0.02 (0.03)	0.03 (0.03)	−0.01(0.03)	−0.02(0.03)	−0.05(0.03)	0.01 (0.03)
P1 compassionate attitudes	0.08(0.04)*	0.07(0.04)*	0.14(0.04)***	0.16(0.04)***	0.05 (0.04)	0.17(0.04)***	0.01 (0.04)	0.01 (0.04)	0.09(0.04)*	0.01(0.04)**	−0.01(0.04)	0.02 (0.04)
P1 compassionate behaviors	0.10(0.03)**	0.14(0.03)***	0.11(0.03)***	0.22(0.03)***	0.19(0.03)***	0.05 (0.03)	0.08(0.04)*	0.11(0.04)**	0.02 (0.04)	0.11(0.03)**	0.06 (0.04)^+^	0.00 (0.03)
P2 mindfulness	0.05 (0.03)^+^	0.07(0.03)*	0.01(0.03)	0.01 (0.03)	−0.02(0.03)	0.05 (0.03)	0.05 (0.03)^+^	0.13(0.03)***	0.03 (0.03)	0.07(0.03)*	−0.02(0.03)	0.17(0.03)***
P2 compassionate attitudes	0.04 (0.04)	0.05(0.04)	0.01 (0.04)	0.11(0.04)***	0.02 (0.04)	0.07 (0.04)^+^	0.11(0.04)**	0.12(0.04)**	0.10(0.04)*	0.18(0.04)***	0.04 (0.04)	0.25(0.04)***
P2 compassionate behaviors	0.01 (0.04)	0.04 (0.04)	0.05 (0.04)	0.01(0.04)	0.03 (0.04)	−0.05(0.04)	0.08 (0.04)	0.08(0.04)*	0.03 (0.04)	0.16(0.04)***	0.24(0.04)***	0.05 (0.04)
R-squared values for each outcome	0.06(0.01)***	0.10(0.02)***	0.08(0.01)***	0.19(0.02)***	0.06(0.01)***	0.14(0.02)***	0.06(0.01)***	0.11(0.02)***	0.04(0.01)**	0.16(0.02)***	0.09(0.02)***	0.15(0.02)***
**Longitudinal relations (T2 outcomes)**												
P1 mindfulness	0.06 (0.03)^+^	0.05 (0.03)	0.03 (0.02)	0.03 (0.03)	0.02(0.03)	0.06(0.03)*	0.03 (0.03)	−0.03(0.03)	0.00 (0.02)	0.01 (0.03)	0.00 (0.03)	0.04 (0.03)
P1 compassionate attitudes	−0.03(0.04)	−0.04(0.04)	0.01 (0.03)	0.11(.04)**	0.02 (0.04)	0.08(0.04)*	−0.01(0.04)	−0.01(0.04)	0.06 (0.04)	0.04 (0.04)	0.04 (0.03)	0.03 (0.04)
P1 compassionate behaviors	0.03 (0.03)	0.08 (0.04)	−0.04(0.03)	0.07(0.03)*	0.11(0.03)**	0.05 (0.03)	0.06 (0.03)	0.12(0.04)**	0.01 (0.03)	0.02 (0.04)	–0.06 (0.04)^+^	−0.01(0.04)
P2 mindfulness	0.00 (0.03)	0.04 (0.03)	−0.03(0.03)	−0.02(0.03)	0.04 (0.03)	0.00 (0.03)	0.05 (0.03)	0.08(0.03)**	0.01 (0.03)	0.10(0.03)**	0.06(0.03)*	−0.03(0.03)
P2 compassionate attitudes	0.07 (0.04)	−0.04(0.04)	0.01 (0.03)	0.04 (0.04)	0.00 (0.03)	0.06 (0.04)	0.06 (0.04)	0.02 (0.04)	0.05 (0.04)	0.11(0.04)*	−0.01(0.04)	0.10(0.05)*
P2 compassionate behaviors	0.04 (0.04)	0.08(0.04)*	0.02 (0.03)	0.09(0.04)*	−0.04(0.04)	−0.04(0.04)	0.01 (0.04)	0.02 (0.04)	0.01 (0.04)	0.09(0.04)*	0.13(0.04)**	0.05 (0.04)
R-squared values for each outcome	0.17(0.02)***	0.16(0.02)***	0.43(0.03)***	0.20(0.02)***	0.22(0.02)***	0.26(0.02)***	0.17(0.02)***	0.19(0.02)***	0.26(0.04)***	0.21(0.02)***	0.20(0.02)***	0.19(0.03)***

R-square values for each outcome at each time point. Longitudinal effects control for stability in constructs across 2 years. Controls included race, age, and education where relevant (parameter estimates unshown).

^+^*p* < 0.10, *p < 0.05, **p < 0.01, ***p < 0.001.

#### Cross-sectional actor associations

Cross-sectionally (T1), the model showed numerous significant associations between relationally compassionate constructs and sexual well-being for wives. Indeed, *mindfulness* was positively related to one’s own sexual satisfaction (β = 0.10, SD = 0.03, *p* < 0.001), orgasm consistency (β = 0.06, SD = 0.03, *p* = 0.04), and sexual non-judgment (β = 0.21, SD = 0.03, *p* < 0.001). Similarly, wives’ compassionate relational *attitudes* related positively to sexual frequency (β = 0.08, SD = 0.04, *p* = 0.03) sexual satisfaction (β = 0.07, SD = 0.04, *p* = 0.03), orgasm consistency (β = 0.14, SD = 0.04, *p* < 0.001), sexual harmony (β = 0.16, SD = 0.04, *p* < 0.001), and non-judgment (β = 0.17, SD = 0.04, *p* < 0.001). Compassionate relational *behaviors* was the only construct to relate to sexual awareness, in the positive direction (β = 0.19, SD = 0.03, *p* < 0.001), in addition to positively relating to sexual frequency (β = 0.10, SD = 0.03, *p* < 0.01), sexual satisfaction (β = 0.14, SD = 0.03, *p* < 0.001), orgasm consistency (β = 0.11, SD = 0.03, *p* < 0.01), and sexual harmony (β = 0.22, SD = 0.03, *p* < 0.001).

Husbands showed significant positive relations between all three aspects of relational compassion and their own sexual well-being cross sectionally. Husbands’ *mindfulness* was positively related to their own sexual satisfaction (β = 0.13, SD = 0.03, *p* < 0.001), sexual harmony (β = 0.07, SD = 0.03, *p* = 0.02), and non-judgment (β = 0.17, SD = 0.03, *p* < 0.001). Husbands’ relationally compassionate *attitudes* were positively related to sexual frequency (β = 0.11, SD = 0.04, *p* < 0.01), sexual satisfaction (β = 0.12, SD = 0.04, *p* < 0.01), orgasm consistency (β = 0.10, SD = 0.04, *p* = 0.01), sexual harmony (β = 0.18, SD = 0.04, *p* < 0.001), and non-judgment (β = 0.25, SD = 0.04, *p* < 0.001). In slight contrast, husbands’ relationally compassionate *behaviors* were positively related to sexual satisfaction (β = 0.08, SD = 0.04, *p* = 0.046), sexual harmony (β = 0.16, SD = 0.04, *p* < 0.001), and sexual awareness (β = 0.24, SD = 0.04, *p* < 0.001), but not sexual frequency, orgasm consistency or non-judgment.

#### Cross-sectional partner associations

Both wives and husbands showed significant relations between their own compassionate predictors and their partner’s sexual well-being outcomes at T1. Specifically, wives’ relationally compassionate *behaviors* related positively to husbands’ sexual frequency (β = 0.08, SD = 0.04, *p* = 0.03), sexual satisfaction (β = 0.11, SD = 0.04, *p* < 0.01), and harmony (β = 0.11, SD = 0.03, *p* < 0.01). Wives’ *Mindfulness* showed no significant relations, however wives’ relationally compassionate *attitudes* showed one positive significant relation with husbands’ orgasm consistency (β = 0.09, SD = 0.04, *p* = 0.04).

In contrast, husbands’ *mindfulness* positively related to wives’ sexual satisfaction (β = 0.07, SD = 0.03, *p* = 0.01) and their compassionate relational *attitudes* related positively to wives’ sexual harmony (β = 0.11, SD = 0.04, *p* < 0.01). There were no significant relations between husbands’ relationally compassionate *behaviors* and their wives’ sexual well-being.

#### Longitudinal actor associations

Among wives, relations between T1 relational compassion constructs and T2 sexual well-being showed that their *mindfulness* marginally predicted their own sexual frequency (β = 0.06, SD = 0.03, *p* = 0.06) and sexual non-judgment two years later (β = 0.06, SD = 0.03, *p* = 0.03). Further, wives’ compassionate relational *attitudes* were positively related to their own sexual harmony (β = 0.11, SD = 0.04, *p* < 0.01) in addition to their own sexual non-judgment (β = 0.08, SD = 0.04, *p* = 0.03). Finally, relationally compassionate *behaviors* were positively associated with sexual harmony (β = 0.07, SD = 0.03, *p* = 0.04) and sexual awareness (β = 0.11, SD = 0.03, *p* < 0.01).

Husbands showed similar relations, although T1 *mindfulness* longitudinally predicted their own sexual harmony (β = 0.10, SD = 0.03, *p* < 0.01) and awareness (β = 0.06, SD = 0.03, *p* = 0.04), in addition to sexual satisfaction (β = 0.08, SD = 0.03, *p* < 0.01). T1 compassionate relational *attitudes* related to T2 sexual harmony (β = 0.11, SD = 0.04, *p* < 0.01) and sexual non-judgment (β = 0.09, SD = 0.05, *p* = 0.04). Compassionate relational *behaviors* were positively associated with both sexual harmony (β = 0.11, SD = 0.04, *p* = 0.01) and sexual awareness (β = 0.13, SD = 0.04, *p* < 0.01).

#### Longitudinal partner associations

For longitudinal partner associations, wives’ relationally compassionate *behaviors* positively predicted husbands’ sexual satisfaction (β = 0.12, SD = 0.04, *p* < 0.01), whereas husbands’ relationally compassionate *behaviors* predicted wives’ sexual harmony (β = 0.09, SD = 0.04, *p* = 0.02), and sexual satisfaction (β = 0.08, SD = 0.04, *p* = 0.04). No significant paths emerged for either wives’ or husbands’ T1 *mindfulness* or relationally compassionate *attitudes* on their partner’s T2 sexual well-being outcomes.

## Discussion

Sexuality is emotional, physical, and relational in nature (Busby et al., 2022), and represents one of the most intimate and vulnerable acts preformed between romantic partners. Indeed, sexuality not only involves physical nakedness, but high-quality sex requires partners to display “emotional nakedness” as well, being willing to be exposed, accessible and nurtured in a safe space. We build on the DMMC and a multifaceted understanding of compassionate processes (Goetz and Simon-Thomas, 2017; Allsop et al., 2021) to show that relational compassion may be a key precursor to sexually bonding experiences as it conveys trust, care, and acceptance. For the present study, we operationalized *relational compassion* using a variety of interrelated measures that capture elements of compassion (i.e., mindfulness, relational attitudes and behaviors)—and found that it may facilitate deeper understanding and connection for newly married couples trying to maintain an intimate relationship through sexual expression. We wanted to explore how specific elements of relational compassion bring immediate links to sexual well-being and what elements may have longer lasting benefits as sex is important in the moment, but can also help sustain a high-quality, lasting relationship (Busby et al., 2022). We discuss the results in terms of the cross-sectional findings and the two-wave time period findings below.

### Cross-sectional associations between compassion and sexual well-being

As wives and husbands reported greater relational compassion, we found several positive connections for both actor and partner. Mindfulness and compassionate attitudes (accessibility, responsiveness, and engagement) were connected to most of the individuals’ own sexual well-being markers, but neither was associated with own sexual mindful awareness. This finding is not entirely surprising as sexual mindfulness is more difficult to achieve than simple trait mindfulness due to heightened anxiety and self-evaluations during sexual encounters (Leavitt et al., 2019). This finding shows that trait mindfulness may be necessary, but not sufficient to achieving relational, sexual mindfulness. Findings prompt future study into how partners can practice and incorporate sexual mindfulness into their lives given its positive correlates in previous research (Leavitt et al., 2021c). Compassionate behaviors (forgiveness and gratitude), however, *were* connected to all of the individuals’ sexual well-being markers at T1. These findings are aligned with previous research as well as the DMMC (Allsop et al., 2021) and the relational mindfulness framework (Karremans et al., 2017). It may be that in an immediate way, compassionate behaviors represent a potent balm for relationships, with forgiveness and gratitude (within the relationship context) prompting individuals be more introspective and connected to their partner, which in turn facilitates individual sexual behaviors and well-being. More than just alleviating distress (Santerre-Baillargeon et al., 2018; Michael et al., 2021), compassionate behaviors provide targeted and supportive emotional and relational comfort to the self (Germer, 2009), which then encourages an accepting sexual environment (Leavitt et al., 2021e).

In addition to individual outcomes, we found that an individual’s mindfulness and compassionate attitudes and behaviors were also connected to their *partner’s* sexual well-being. Wives’ relationally compassionate behaviors, but not their mindfulness or compassionate attitudes, were associated with multiple markers of their husbands’ sexual well-being. Husbands may be particularly sensitive to compassionate behaviors of forgiveness and gratitude, which are acts of other-connectedness (Karremans et al., 2017; Allsop et al., 2021). Previous research has shown that forgiveness and gratitude are two ways that mindfulness may work to benefit couples’ relational and sexual well-being because they open up lines of communication, prompt perspective taking, and help partners work through challenges (Goetz and Simon-Thomas, 2017; Eyring et al., 2021). The present findings support this work, showing that particularly for husbands, their partner’s willingness to remain committed and engaged despite faults can enhance their sexual well-being. Wives’ gratitude was additionally important, perhaps signifying how a husband’s sexual well-being can be sustained through feeling that they are appreciated and meeting their wife’s physical and emotional needs. Such positive psychological thoughts around acceptance and appreciation may help husbands more fully enjoy the physical closeness inherent to sexual experiences (Allsop et al., 2021).

Husbands’ mindfulness and relationally compassionate attitudes were particularly important for their wives’ sexual well-being. Husbands’ higher mindfulness was associated with wives’ higher sexual frequency and sexual satisfaction. Additionally, relationally compassionate attitudes of accessibility, responsiveness, and engagement were associated with feelings of harmony within the sexual relationship as well as feeling less judgmental during sex. It could be that as men focus on awareness and on bonding with their partner in deep emotional ways, they create an environment conducive to physical intimacy where their wife is comfortable seeking physical intimacy. Additionally, men’s compassionate attitudes may create more other-centeredness and other-connectedness that enables wives’ sexual well-being via feelings of acceptance, confidence, certainty and love despite personal insecurities (Johnson, 2003; Allsop et al., 2021). These compassionate attitudes may create critical bonding moments (Sandberg et al., 2012) that help women in particular feel more connection and thereby experience a more enjoyable, vibrant sexual interaction. It is also possible that husbands’ close attention (i.e., mindfulness) to sensation enhances wives’ sexual experience, which then promotes greater frequency because past experiences were positive.

These findings highlight how husbands’ accessibility and responsiveness might create a safe space for wives to feel more aligned with their partner (i.e., sexual harmony) and tuned-in to the experience rather than being distracted or depreciated by self-judgment. Future work should explore these relations more thoroughly to understand reciprocity and contextual elements around sexual relationships, particularly how husbands can create safe, nurturing environments for wives to express sexual needs.

### Links over time between compassion and sexual well-being

The two-wave findings in this study were not as pronounced as the cross-sectional associations, but this is not entirely unexpected. There may be a number of temporal effects of relational compassion on sexuality. Mindfulness, along with compassionate attitudes and behaviors may be more effective at the time when they are demonstrated and may not necessarily carry over long periods of time (Eyring et al., 2021; Smedley et al., 2021). However, we did find some associations across time periods. For the individual, wives’ mindfulness significantly predicted their own sexual non-judgment two years later. This is likely due to the ability to be fully present in the moment, enjoying sexual sensations. This may create a pattern of focusing on pleasurable sensations and not ruminating on one’s performance or physical appearance during sex, which facilitates non-judgment over time. Conversely, wives’ compassionate relational attitudes predicted their own sexual harmony and non-judgment and relationally compassionate behaviors predicted their own sexual harmony and awareness two years later. In contrast to personal mindfulness, it appeared that more relational elements of compassion, including other-centeredness and other-connectedness, acceptance and nurturance of their partner led to wives’ own psychological connection to their sexuality, which in turn bonds them to their partner. Indeed, earlier mindsets that focus on personal sensation as well as relational connection may set a tone for later sexual acceptance and connection (Karremans et al., 2017).

Husbands showed similar associations: mindfulness predicted their own sexual harmony, awareness and sexual satisfaction two years later. Husbands’ compassionate relational attitudes predicted sexual harmony and non-judgment, and compassionate relational behaviors predicted their own harmony and awareness two years later. The findings regarding sexual awareness are particularly positive, as more sexual mindful awareness is certainly associated with a better sexual well-being overall (e.g., Leavitt et al., 2020, 2021c). Karremans et al. (2017) framework explains that increased mindful awareness, and we would argue relational compassion, may facilitate a better understanding of how other’s behaviors are impacted by external circumstances (Block-Lerner et al., 2007). Therefore, as men increase their own mindfulness and overall relational compassion, the more they might understand how their wife’s sexual behavior may be impacted by things she cannot control (e.g., stressful work environment, parenting, etc.). Studies also suggest that mindfulness (we would include relational compassion) can increase empathy, understanding, gratitude and even forgiveness of a spouse’s actions (Block-Lerner et al., 2007; Birnie et al., 2010; Eyring et al., 2021). These compassionate skills likely increase feelings of interconnectedness (Brown and Ryan, 2003), which will increase sexual well-being, as evidenced in our model.

Partner effects across time periods showed that for wives, relationally compassionate behaviors positively predicted husbands’ sexual satisfaction (and sexual awareness marginally), whereas husbands’ relationally compassionate behaviors predicted wives’ sexual harmony and sexual satisfaction two years later. Surprisingly, mindfulness and relationally compassionate attitudes were not associated across time periods to sexual well-being outcomes. These findings should be further investigated. It may be that some elements of compassion are only immediately impactful as described by framework Karremans et al. (2017) and may not have long lasting impact. For example, mindfulness is concerned with being fully present in a particular moment. Additionally, being accessible and responsive at one time point may not have lasting effects as circumstances change and partners may not have the capacity to always respond in this way. Indeed, partners may need to consciously work to have relationally compassionate attitudes at all times to see lasting effects, particularly in the early years of a marriage. It may be that the power of compassion is immediate connection and benefits are fleeting. However, we find it interesting that compassionate behaviors carried the most weight in the model across time periods for both partners. This finding underscores the importance of gratitude and forgiveness in maintaining the kind of relationship that can enable lasting sexual satisfaction and harmony. A significant body of literature has shown the positive benefits of both forgiveness and gratitude to marriage quality and length (Fincham et al., 2006; Gordon et al., 2011). Our study corresponds with this work, but furthers it by showing relationally compassionate behaviors contribute to *sexual* well-being in marriages specifically. Understanding what elements of compassion are short-lived and what elements may have more lasting effects is salient for couples, therapists, and educators in promoting high-quality sex and overall relationships. In the present study, we found that mindfulness and compassionate attitudes of awareness, responsiveness and engagement should be fostered for short-term benefits, but lasting effects may be most supported by promoting compassionate behaviors of forgiveness and gratitude. Both the short term and long-term benefits are likely due to the other-connectedness that compassion provides (Carroll et al., 2006; Goetz and Simon-Thomas, 2017; Allsop et al., 2021). It is likely that forgiveness and gratitude are related to future mindfulness and compassionate attitudes, which subsequently relate to sexual well-being in a mediational way. We hope to see these associations tested in the future. However, at the time being we advise individuals and relationship educators to prize relational compassion, and specifically forgiveness and gratitude, as tangible skills for promoting healthy sexuality.

### Strengths and limitations

This research on relational compassion is preliminary and needs further exploration. Our data came from a US nationally representative longitudinal study. Although we only used two waves of the longitudinal date, we were able to measure cross-sectional and longitudinal effects across two years. All participants in our sample were newly married and primarily in their 20s and 30s; thus, conclusions are generalizable only to this subgroup. This is both a strength and a limitation as additional research will need to clarify whether these findings apply to couples in other demographic categories. We cannot rule out changes in relationships due to maturation, historic events, or repeated testing and note that we did not have a “total length of relationship before marriage” variable to be used as a control. Future research could examine how relationally compassionate elements link to sexual well-being in couples in mid- and late life relationships, same-sex relationships, across various relationship types, lengths and cohabitation practices, and other important demographic groups.

We also suggest that future researchers continue to expand the way that compassion might be measured within relationships. Although we consulted many definitions of compassion, (e.g., Goetz and Simon-Thomas, 2017), and crafted a relational framework that included mindfulness, attitudes, and behaviors, there may be other ways to conceptualize compassion (i.e., prosociality, targeted sympathy, intimate knowledge) that are worth exploring. Similarly, although we present a fairly robust battery of measures of sexual well-being, we recognize that some factors are likely left-out, including more comprehensive measures of sexual satisfaction. Our study represents a first foray into the relations between compassion and sex within relationships, and we hope that future work builds on this study to explore mediators, moderators, and potential transactional relations between constructs over time in pursuit of deeper understanding on what relational compassion looks like and how it operates to strengthen and sustain relationships through healthy sexuality.

Finally, we note that participants were each expressly asked to take the survey independently from their spouse and were provided different survey links. However, we cannot be positive that every participant adhered to these instructions, which could compromise validity. Future research could take more precautions against self-report bias as well as partner influence during the survey.

## Conclusion

Despite limited effects across waves of data, this study showed that women’s sexual well-being was driven cross-sectionally by husbands’ relationally compassionate attitudes (accessibility, responsiveness, and engagement) and across two time periods by husbands’ relationally compassionate behaviors (forgiveness and gratitude), whereas husbands’ sexual well-being was driven both cross-sectionally and across the two time periods by wives’ relationally compassionate behaviors of forgiveness and gratitude. Results provide initial evidence of how compassionate behaviors, particularly for women, can enhance and sustain their sexual connection with their partner within the moment. In contrast, compassionate behaviors from both partners had lasting effects on sexual satisfaction and women’s perceptions of sexual harmony even two years later. In this article, we show how compassion can not only help individuals, but can also be conceptualized as a relational construct that enhances marriages, partnerships, and sexuality. Indeed, relational compassion, and particularly relationally compassionate *behaviors*, may be a key facilitator of sexual well-being, particularly for newly married couples.

## Data availability statement

The original contributions presented in this study are included in the article/supplementary material, further inquiries can be directed to the corresponding author.

## Ethics statement

The studies involving human participants were reviewed and approved by Brigham Young University Internal Review Board. The patients/participants provided their written informed consent to participate in this study.

## Author contributions

AF, CL, and JY conceptualized the theoretical model. AF and JY undertook analyses and wrote Method and Results. CL and AP primarily wrote the literature review. All authors contributed to the Discussion, editing, and formatting.

## Footnotes

1 Sex is a broad term and includes a wide range of activities. We are not directive about what activities participants consider “sex,” likely resulting in sex and sexual well-being being more inclusive, which is supported by other research on sexual well-being (Leavitt et al., 2021d; Waite et al., 2017).
